# A Comprehensive Review of the Pharmacological Properties and Bioactive Components of *Retama monosperma*

**DOI:** 10.3390/molecules28041708

**Published:** 2023-02-10

**Authors:** Adil El Yadini, Youssef Elouafy, Ehsan Amiri-Ardekani, Mina Shafiee, Amirhosein Firouzi, Najmeh Sasani, Asaad Khalid, Ashraf N. Abdalla, Saad Bakrim, Ching Siang Tan, Khang Wen Goh, Long Chiau Ming, Abdelhakim Bouyahya

**Affiliations:** 1 Laboratory of Materials, Nanotechnology and Environment LMNE, Faculty of Sciences, Mohammed V University in Rabat, Rabat BP 1014, Morocco; 2 Department of Phytopharmaceuticals (Traditional Pharmacy), Faculty of Pharmacy, Shiraz University of Medical Sciences, Shiraz P.O. Box 64685-71468, Iran; 3 Research Center for Traditional Medicine and History of Medicine, Shiraz University of Medical Sciences, Shiraz P.O. Box 64685-71468, Iran; 4 Center for Nanotechnology in Drug Delivery, School of Pharmacy, Shiraz University of Medical Sciences, Shiraz P.O. Box 64685-71468, Iran; 5 Essential Oils Research Institute, University of Kashan, Kashan P.O. Box 87516-51998, Iran; 6 Nutrition Research Center, School of Nutrition and Food Sciences, Shiraz University of Medical Sciences, Shiraz P.O. Box 64685-71468, Iran; 7 Student Research Committee, Shiraz University of Medical Sciences, Shiraz P.O. Box 64685-71468, Iran; 8 Substance Abuse and Toxicology Research Center, Jazan University, Jazan 45142, Saudi Arabia; 9 Medicinal and Aromatic Plants and Traditional Medicine Research Institute, National Center for Research, Khartoum P.O. Box 2404, Sudan; 10 Department of Pharmacology and Toxicology, College of Pharmacy, Umm Al-Qura University, Makkah 21955, Saudi Arabia; 11 Geo-Bio-Environment Engineering and Innovation Laboratory, Molecular Engineering, Biotechnologies, and Innovation Team, Polydisciplinary Faculty of Taroudant, Ibn Zohr University, Agadir 80000, Morocco; 12 School of Pharmacy, KPJ Healthcare University College, Nilai 71800, Malaysia; 13 Faculty of Data Science and Information Technology, INTI International University, Nilai 71800, Malaysia; 14 School of Medical and Life Sciences, Sunway University, Sunway City 47500, Malaysia; 15 Laboratory of Human Pathologies Biology, Department of Biology, Faculty of Sciences, Mohammed V University in Rabat, Rabat 10106, Morocco

**Keywords:** *Retama monosperma* L., medicinal plant, isolated compounds, pharmacological properties, bioactive components

## Abstract

*Retama monosperma* L. (Boiss.) or *Genista monosperma* L. (Lam.), known locally as “R’tam”, is a spontaneous and annual herb that belongs to the Fabaceae family. It is native to the Mediterranean regions, specifically in the desert areas and across the Middle Atlas in Morocco. This plant has been extensively used in folk medicine and it is rich in bioactive compounds, including polyphenols, flavonoids, and alkaloids. Current research efforts are focusing on the development of novel natural drugs as alternatives to various organic and non-organic chemical products from *Retama monosperma*. In addition, extract, and isolated compounds obtained from different parts of the chosen plant have been described to exhibit multiple biological and pharmacological properties such as antioxidant, anti-aging, anti-inflammatory, antihypertensive, anti-helminthic, disinfectant, diuretic, and hypoglycemic effects. The plant-derived extract also acts as an antimicrobial agent, which is highly efficient in the treatment of bacterial, viral, and fungal infections. Its antiproliferative effects are associated with some mechanisms, such as the inhibition of cell cycle arrest and apoptosis. In light of these assessments, we critically highlight the beneficial effects of the flowers, stems, seeds extracts, and isolated compounds from *R. monosperma* (L.) Boiss in human health care, industrial, and other applications, as well as the possible ways to be employed as a potential natural source for future drug discovery.

## 1. Introduction

*Retama monosperma* L. (Boiss.) or *Genista monosperma* L. (Lam.), known locally in the popular Arabic name as “R’tam” [1], is a spontaneous, abundant, and annual herb that belongs to the family of Fabaceae. It is endemic to the west of the Mediterranean basin, such as the Canary Islands, Portugal, Italy, southwest Spain, Macaronesia, North Africa, and northern Egypt [2]. In Morocco, it is situated in the desert areas and across the Middle Atlas and in several Moroccan natural forests [3]. It has a large geographic distribution and represents a potential for use in the stability of dunes and the revegetation of desert ecosystems, and is occasionally cultivated as an ornamental herb, especially in Mediterranean climates [4]. For many years, plants have been used by humans for medicinal and nutritional purposes in the food industry and other applications [5,6,7,8]. In recent decades, they have drawn significant interest and represent a largely untapped source of novel and effective drugs to overcome resistance to the treatment of a wide spectrum of diseases or to be used as alternatives to different organic and non-organic chemical products due to their significant bioactivities [9,10]. In Morocco, medicinal herbs have always been linked to both traditional and cultural practices [11,12].

*R. monosperma* is considered one of the medicinal plants rich in bioactive compounds, including alkaloids, polyphenols, flavonoids, fatty acids, and condensed tannins, as proved by several studies [3,13,14,15,16]. It has been extensively used in folk medicine in a wide range of countries; for example, used as an effective antihelmintic, disinfectant, and abortifacient and also to treat skin damages and cicatrization [3]. 

*R. monosperma* has attracted considerable interest due to its wide range of pharmacological properties, including antioxidant [17], anti-aging [3], antibacterial [18], antifungal [19], anti-inflammatory, antiproliferative, and antitumoral [3,11,20], as well as antileukemic activities [21]. The principal alkaloids of *R. monosperma* are retamine, sparteine, dehydrosparteine, ammodendrine, N-methylcytisine, cytisine, isolupanine, and anagyrine [22], flavonoids such as genistein, quercetin, 6-methoxykaempferol, and kaempferol [16], fatty acids from the seeds’ hexane extract, such as myristic, pentadecylic, oleic (omega-9), linoleic (omega-6) and linolenic (omega-3) [23]. It was found that *R. monosperma* exhibited an anti-aging effect that could enhance the expression of genes that play a role in wound healing and skin regeneration, such as sirtuin 1 (SIRT1) and SIRT3 in the HaCaT human keratinocyte cell line [3]. In addition, this plant showed potent antimicrobial effects and could be used to formulate medicinal plants for the management of various infectious conditions. It has been reported that hexane and dichloromethane extracts of seeds showed a very significant (Ø > 14 mm) antibacterial effect against *Bacillus sp* and *E. coli*, while the ethyl acetate extracts of the stems and the flowers exhibited very significant activity on *Salmonella sp* [18]. As an antifungal activity, alkaloids of *R. monosperma* play a significant role in the reduction of *C. albicans* and *C. tropicalis* growth. Furthermore, previous investigations have proven that *R. monosperma* has a promising anti-inflammatory effect; it is suggested that this species could be an option for developing an herbal medicine for inflammatory bowel disease due to its ability to reduce the production of pro-inflammatory cytokines such as COX-2 and iNOS [23]. Moreover, the findings of certain investigations have demonstrated that *R. monosperma* extracts showed beneficial antitumoral effects on human cervical adenocarcinoma cell lines (HeLa and SiHa) proliferation and apoptosis [3]. In addition to this, *R. monosperma* can be a candidate for traditional use as an antileukemic plant because it has been revealed to contain several unsaturated fatty acids, particularly linoleic acid, which is considered to be beneficial in cancer [24]. Concerning the antioxidant activity of *R. monosperma*, it has been observed to exert powerful effects against oxidative stress-mediated pathological processes, which are attributed to the presence of flavonoids in ethyl acetate extracts of seeds, more specifically [17]. The literature in the previous year’s reports contains a number of reviews on the phytochemistry and biological functions of several bioactive compounds of different parts of *R. monosperma*, but comprehensive investigations focusing on their health benefits are missing. This calls for further coordination on the state of knowledge to analyze the full potential pharmacological effects of the main bioactive compounds of different parts of this Mediterranean plant to better understand its benefits to human health and explore its clinical applications and pharmaceutical industries.

The aim of this manuscript is to emphasize the potential pharmacological activity of *R. monosperma* and its classification of bioactive components in modern pharmacological research.

## 2. Chemical Composition 

The chemical compounds of *R. monosperma* are rich in bioactive constituents belonging to different chemical classes, such as terpenoids, flavonoids, phenolic acids, fatty acids, and alkaloids. Table 1 shows the chemical composition of *R. monosperma* according to plant parts. 

### 2.1. Polyphenols, Flavonoids, and Tannins

**Figure 1:**
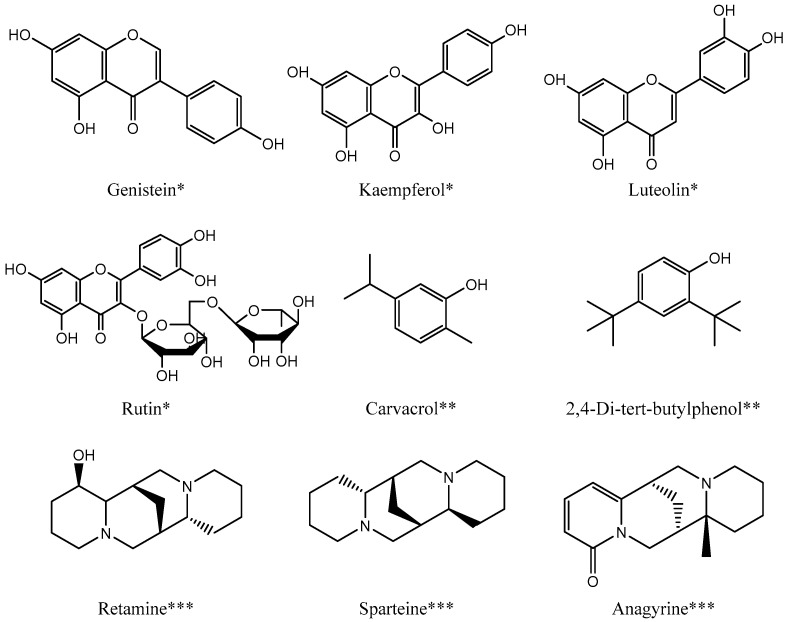
Some chemical structures of flavonoids (*), phenols (**), and alkaloids (***) were isolated from *Retama monosperma*.

Polyphenols, flavonoids, and tannins, which are organic compounds found abundantly in plants, have become an emerging field of interest in nutrition in recent decades. *Retama monosperma* can be considered one of the plants rich in those compounds, as proven by several studies [3,13,14,15,16]. Previous studies have aimed to identify specific compounds present in *R. monosperma* through various identification methods [16,29]. Conversely, other authors have chosen to qualitatively quantify the content of certain groups of molecules, such as polyphenols, flavonoids, and condensed tannins, without identifying each individual compound [13,14]. Belmokhtar et al., 2014 reported that ethyl acetate extract has the highest polyphenol content in any of the stems, flowers, and seeds [13], while Hamdani et al. recorded in 2018 that the highest total polyphenols contents were reported in the methanol extract of the stems and the methanolic extract of the flowers showed a higher tannins content than the other extracts [14]. In 2021, Selaimia et al. successfully identified several compounds present in *Retama monosperma* using dried and crushed leaves as the source material [29]. The compounds were identified using GC/MS analysis. Among the compounds identified were two phenol compounds, carvacrol, which was found in the butanol extract, and 2,4-di-tert-butylphenol (Figure 1), which was identified in the ethyl acetate extract [29]. Earlier in 2013, González-Mauraza et al. identified seven flavonoid molecules in the aqueous extract of *Retama monosperma*, and these molecules are daidzin, rutin, genistin, daidzein, luteolin, and apigenin, and the main flavonoid was genistein [16].

### 2.2. Alkaloids

Alkaloids are a diversified structural group of natural products, and these molecules have a vast array of biological activities; many of them have significant pharmacological applications. In 1980, Antonio Salatino and Otto R. Gottlieb reported the presence of five quinolizidine alkaloids by GLC-MS in *Retama monosperma,* and these alkaloids are retamine, sparteine, anagyrine, cytisine and N-methylcytisine [22]. In addition to these molecules, A. El-Shazly et al. isolated four other alkaloids in 1996, including ammodendrine, 17-oxosparteine, lupanine and 5,6-dehydrolupanine, from stems and seeds of *Retama monosperma* using CLC and GLC-MS [19]. 

Another study was conducted by N. Merghoub et al. in 2011 on the dichloromethane fraction of the *Retama monosperma* leaves, and the results led to revealed five known quinolizidine alkaloids as well as sparteine, L-methylcytisine, 17-oxosparteine, and lupanine and anagyrine as a major alkaloid (Figure 1) [30]. In 2012, a study by Fdil et al. showed the presence of these alkaloids in the three parts of the methanolic extract of *Retama monosperma* (stems, leaves and seeds) with the identification of four new alkaloids, which are dehydrosparteine and isolupanine in the stems and leaves, dehydrocytisine in the seeds and β-isolupanine in the leaves [25], and since then, several investigations have been carried out showing the presence of these alkaloids with different percentages depending on the part of the plant and/or the solvent used during extraction [15,17,18,26,29].

It is noted that in most cases, the seeds contain mainly cytisine, while in the stems and leaves, sparteine and anagyrine are the predominant alkaloids.

### 2.3. Fatty Acids

The fatty acids present in the lipid and hexane seeds and cladodes extracts of *R. monosperma* were analyzed using GC/MS. The results of the analysis are presented in Table 1, which shows the presence of oleic acid (Omega 9), linoleic acid (Omega 6), linolenic acid (Omega 3), arachidic acid, myristic acid, pentadecylic acid, palmitic acid, palmitoleic acid, margaric acid, stearic acid, behenic acid, lignoceric acid, lauric acid, tricosanoic acid, palmitoleic acid, and pentadecanoic acid [2,21,23,28].

## 3. Biological Properties

Different investigations showed that *R. monosperma* exhibits several biological and pharmacological properties. All these effects are presented in the flowing table (Table 2) and extensively discussed. 

### 3.1. Antioxidant and Anti-Aging Activity

Normal metabolism and cellular respiration resulted in producing reactive oxygen species (ROS), which are highly active molecules associated with physiological and pathological processes. Oxidative stress damages cells and tissues and causes several diseases when the balance between the accumulation of ROS and the body’s antioxidant process is disturbed [33]. On the other hand, antioxidants had a defensive function against oxidative stress-mediated pathological processes [34]. Herbal compounds especially polyphenols such as phenolic acids, flavonoids, tannins, anthocyanins, are known for their free radical scavenging and antioxidant activities [35].

Certain studies showed the antioxidant effects of *R. monosperma.* Indeed, Belmokhtar et al. examined the antioxidant activity of the various hydromethanolic extract (chloroform, ethyl acetate, toluene, and butanol) of the flowers, seeds, and stems of *R. monosperma.* They utilized 2,2-diphenyl-1-picrylhydrazyl (DPPH) radical scavenging assay and compared results to ascorbic acid as a control sample [13]. The results indicated that there was no relationship between condensed tannin and antioxidant activities, but the potential source of the antioxidant power of *R. monosperma* is related to flavonoids in ethyl acetate extracts of seeds, (IC_50_ = 0.15 ± 0.11 mg/mL) [13].

In addition, Zefzoufi et al. evaluated the antioxidant activity of ethyl acetate extract of seeds (EAS) and diethyl ether extract of flowers (DEF) and their isolated compounds using DPPH assay. The main isolated compounds of EAS were quercetin, 6-methoxykaempferol and kaempferol and for DEF was genistein. They reported that the antiradical activities of EAS, 6-methoxykaempferol and kaempferol were significantly greater than quercetin and BHT (*p* < 0.05) (EAS IC_50_ = 15.13 μg/mL compared to BHT IC_50_ = 30.21 μg/mL); however, the DEF and genistein showed moderate antiradical activity when compared with BHT [3]. On the other hand, they claimed that the seeds and flower extract of *R. monosperma* and the isolated flavonoids (genistein, quercetin, 6-methoxykaempferol and kaempferol) have an anti-aging effect that could enhance the expression of genes that play a role in wound healing and skin regeneration, such as sirtuin 1 (SIRT1) and SIRT3 in the HaCaT human keratinocyte cell line [3].

### 3.2. Antimicrobial Activity

Several studies showed that plant-derived antimicrobial substances are highly efficient in the treatment of bacterial, viral, and fungal infections [36]. Here, we bring the studies that evaluated the antimicrobial activity of *R. monosperma.*

The rising in antibiotic-resistant bacteria is a reason for developing new antibacterial agents. Belayachi et al. designed a study to determine total polyphenols, flavonoids and tannins contents in ethyl acetate, hexane, methanol, and dichloromethane extracts from aerials parts of *R. monosperma* (seeds, stems, and flowers) and assess their antibacterial activities against various strains of bacteria by disc diffusion method [32]. They demonstrated that the aerials parts of *R. monosperma* extracts have low contents of total flavonoids compared to tannins and total phenols contents. Furthermore, they reported that all of the extracts were inactive against all tested strains at concentrations of 250 and 125 mg/mL, while the concentration of 500 mg/mL showed the highest antibacterial activity in seeds, followed by flowers. The stems showed relatively lower activity [32]. In detail, hexane and dichloromethane extracts of seeds showed a very significant (Ø > 14 mm) antibacterial effect against *Bacillus sp* and *Escherichia coli*, while the ethyl acetate extracts of the stems and the flowers exhibited very significant activity on *Salmonella sp*. They also claimed that several bioactive components of *R. monosperma* aerial parts’ extracts lead to synergistic antibacterial effects [32].

Altogether Belayachi et al. concluded that the antibacterial activity of *R. monosperma* differs based on the part of the plant used and the solvent. Also, they asserted that seeds have the best potential antibacterial effect, followed by flowers and stems, in order to formulated herbal medicine for treating various infectious diseases [32].

In another study, El Hamdani et al. investigated the alkaloids’ methanolic extracts of the *Retama monosperma* flowers, stems, leaves, and seeds for antifungal activity against three human fungal pathogens (*Candida albicans, Candida tropicalis,* and *Aspergillus niger*) in various concentrations (500, 250, 125, 62.5, and 31.25 μg/mL) using the disc diffusion method. The results showed that the alkaloids extract from seeds and flowers have no antifungal activity, but the stems and leaves extracts were dose-dependently active. The maximum antifungal activity was detected in a concentration of 500 μg/mL of leaf alkaloid extract against both *Candida* species, followed by stem alkaloid extract [17].

In addition, El Hamdani et al. declared that quinolizidine alkaloids of *R. monosperma* play a significant role in the reduction of *C. albicans* and *C. tropicalis* growth. In fact, sparteine, ammodendrine, and anagyrine, which are the main component in alkaloids extracts of leaves and stems, might be responsible for their antifungal characteristics. However, the inactivity of alkaloid extracts of seeds might be related to the high percentages of cytisine and its derivatives [17].

### 3.3. Antiproliferative and Antitumoral Activities

Cancer is a significant concern in the world, with high mortality rates. There is an increasing interest in developing new drugs from natural products by recognizing active ingredients that are effective against cancer cells. Noticeably, about 60% of drugs presently utilized for cancer treatment have been isolated from natural products [11,37]. The two important mechanisms against the uncontrolled growth of tumor cells are arresting cell proliferation and induction of apoptosis [38]. There are some studies focused on the antitumoral effect of *R. monosperma*. Merghoub et al. studied the antitumoral effects of different *R. monosperma* extracts on human cervical adenocarcinoma cell lines (HeLa and SiHa) proliferation and apoptosis [30]. The antiproliferative effect was determined using MTT assay. They treated cells with the extracts at different concentrations (5–80 μg/mL) for 72 h and reported that *R. monosperma* dichloromethane fraction (Rm-DF) was the most active extract, providing a significant cytotoxic activity on HeLa and SiHa cells in a dose-dependent manner. Furthermore, the ethyl acetate extract showed weak cytotoxic activity, while hexane and methanolic extracts showed no effect [30].

On the other hand, Merghoub et al. treated HeLa and SiHa cells with Rm-DF for 24, 48, and 72 h and conducted Hoechst 33,342 staining to investigate the association of cell growth inhibition by Rm-DF with apoptosis. They claimed that cells with usual features of apoptosis, such as nuclear chromatin condensation or apoptotic bodies, were observed after treatment [30]. In sum, this in vitro study indicated that Rm-DF has a potential antitumoral effect against cervical cancer cell lines through the inhibition of proliferation and induction of apoptosis.

In another study, Benbacer et al. evaluated the anticancer effects of some medicinal plant extracts on both SiHa and HeLa cells. The cell lines were treated with plant extracts at different concentrations (15 to 500 μg/mL) for 48 h and evaluated by MTT colorimetric assay. They asserted that *R. monosperma* methanolic extract showed high cytotoxic activity with the lowest IC_50_ values (99 ± 1 μg/mL in SiHa cells and 96 ± 4 μg/mL in HeLa cells) [1]. Furthermore, Rm-DF was the most cytotoxic active fraction against SiHa and HeLa cell lines in a dose-dependent manner. The IC_50_ values were 14 ± 4 and 21 ± 7 μg/mL in the SiHa and HeLa cell lines, respectively [11].

On the other hand, Benbacer et al. measured ROS generation in SiHa and HeLa cell lines with a ROS-sensitive fluorescent C2938 probe. They treated the cell lines via Rm-DF (20 μg/mL) for 24 h. The results demonstrated a dose-dependent enhancement in intracellular ROS generation, which could lead to apoptosis via the mitochondrial pathway [11].

Benbacer et al. claimed that Rm-DF has cytotoxic effects against cervical cancer cell lines by the prevention of proliferation and induction of apoptosis through a mitochondria-mediated signaling pathway. Therefore, it could be a potential candidate to become an anticancer drug [11].

In addition, El Hamdani et al. studied another aspect of *R. monosperma* and reported the presence of unsaturated fatty acids, especially oleic, linoleic, and linolenic acids, in the seeds, branches, and leaves of this plant. Based on the scientific fact that linoleic acid is considered to be beneficial in cancer, they claimed that *R. monosperma* could be a candidate for traditional use as an antileukemic plant [23].

### 3.4. Anti-Inflammatory

In addition to the effects above, the study conducted by González-Mauraza et al. in 2013 is the only study that examined the in vivo biological activity of *Retama monosperma* and eventually revealed an anti-inflammatory property [16]. González-Mauraza et al. prepared a standardized aqueous extract from *R. monosperma,* which contains seven flavonoids (flavonol rutin, flavones luteolin and apigenin, and the isoflavones daidzin, genistin, daidzein and genistein) that isoflavone genistein was the major one (57.2 ± 0.3 mg/100 g). Then, they utilized this standardized extract in flavonoids in a murine model of Crohn’s disease [16].

González-Mauraza et al. revealed that *R. monosperma* prescription (9–18 mg/kg) reduced the inflammation and damage in trinitrobenzene sulfonic acid (TNBS)-induced colonic mucosa without significant dose-response. This anti-inflammatory property could be associated with the weakening of neutrophil function and downregulation of both pro-inflammatory COX-2 and iNOS protein expression. In addition, *R. monosperma* prescription could improve the clinical signs, including declining the progress of the colitis, decreasing the weight/length relative ratio of the colon, reducing the weight loss, and diminishing the adhesions between the colon and adjacent organs. This reduction in colon adhesion is known as a valuable feature of *R. monosperma* on the down-regulating of the inflammatory process [16].

Finally, González-Mauraza et al. suggested that *R. monosperma,* by its anti-inflammatory characteristic, could be an option for developing an herbal medicine for inflammatory bowel disease (IBD) [16].

Given the lack of in vivo studies on this plant (no studies have been done to determine the mechanism of action neither for the inti-inflammatory or other biological activities), it is crucial to further investigate and identify the different biologically active molecules present in *Retama monosperma*, which could have potential applications in the field of cancer treatment, oxidative stress, and anti-inflammatory activity, with further research needed to discover and understand the mechanisms of action for these activities. Moreover, pharmacokinetic and toxicological investigations should be investigated to validate the safety and confirm the absorption of *R. monosperma* and their bioactive compounds.

## 4. Conclusions and Perspectives

Here, we report the chemical composition and biological properties of *R. monosperma*. It was shown that this medicinal species is widely used in traditional medicine to treat several diseases, and laboratory investigations demonstrated the biological properties of its extracts and essential oils in vitro and in vivo. Moreover, almost of the reported studies attributed these biological properties to the presence of bioactive compounds in *R. monosperma* extracts and essential oils. The findings showed that *R. monosperma* is an important natural source for the development of natural drugs; however, other important investigations are needed to validate their drugs. Indeed, pharmacokinetic and pharmacodynamic studies should be carried out to determine the availability and the actions of *R. monosperma* bioactive compounds. In addition, toxicological tests are required to validate the safety of this plant and its bioactive molecules.

## Figures and Tables

**Table 1:** Chemical composition of *R. monosperma*.

Part of the Plant	Extract	Major Component	Ref
Stems	Methanol Dichloromethane n-Butanol Ethyl acetate Chloroform	Polyphenols Flavonoids Condensed tannins	[13,14]
	Methanol	Alkaloid: Retamine Sparteine Dehydrosparteine Ammodendrine N-Methylcytisine Cytisine 17-Oxosparteine Isolupanine 5,6-Dehydrolupamine Anagyrine Lupanine	[15,17,18,19,22,25]
Flower	Methanol	Polyphenols Flavonoids Condensed tannins Alkaloids	[13,14]
	Dichloromethane	Polyphenol Flavonoid Tannins	[14]
	Toluene	Polyphenol Flavonoid Condensed tannins	[13]
	Diethyl ether	Polyphenol Flavonoid: Genistein, Taxifolin Quercetin, 6-methoxykaempferol Kaempferol	[3]
Seeds	Methanol	Polyphenols Flavonoids Condensed tannins	[13,14]
		Alkaloid: N-methylcytisine, Dehydro-cytisine, Cytisine, 5,6-Dehydrolupanine, Thermopsine Ammodendrine Anagyrine	[13,15,17,18,19,25,26,27]
	n-Butanol	Polyphenols Flavonoids Condensed tannins	[13]
	Hexane	Fatty Acids: Myristic acid Pentadecylic acid Palmitic acid Palmitoleic acid Margaric acid Stearic acid Oleic acid (Omega 9) Linoleic acid (Omega 6) Linolenic acid (Omega 3) Arachidic acid Behenic acid Lignoceric acid Lauric acid Tricosanoic acid Palmitoleic acid Pentadecanoic acid	[2,21,23,28]
	Dichloromethane	Polyphenols Flavonoids Tannins	[14]
	Ethyl acetate	Polyphenol Flavonoid Taxifolin Genistein, Quercetin, 6-methoxykaempferol Kaempferol Condensed tannins	[3,13,14,15]
		Mineral: Al, Ba, Cd, Cu, Fe, Mg, Pb, Zn, Mn, Ca, K, Na, P	[21,23]
Leaves	Methanol	Alkaloid: Sparteine Dehydrosparteine β−Isosparteine Ammodendrine N-Methylcytisine Cytisine 17-Oxosparteine Isolupanine 5,6-Dehydrolupamine Anagyrine	[15,17,18,25]
	Hexane	α-Linolenic acid Sterols: Campesterol Stigmasterol β-Sitosterol	[4]
	Ethyl acetate	Ethyl palmitate Phenol: (2,4-Ditertbutylphenol)	[29]
	n-Butanol	Methyl palmitate Methyl 7-octadecenoate Alkaloid: (Sparteine) Phenol: (Carvacrol)	[29]
	Dichloromethane	Alkaloid: Anagyrine Sparteine	[30]
Branches	NaOH 4%	Monosaccharides: Rhamnose Arabinose Fucose Xylose Mannose Glucose Galactose Galacturonic Acid Glucuronic Acid	[31]
Whole plant	Aqueous	Flavonoid: Daidzin Rutin Genistin Daidzein Luteolin Apigenin Genisetein	[16]

**Table 2:** Biological properties of *R. monosperma*.

Plant Part	Extraction Type, Yield, and Studied Dose	Design	Active Compounds	Results	Ref
Seeds Flowers	-Flowers air-dried for two weeks and extracted three times by maceration at room temperature for three days in 3 L of n-hexane -Maceration with 3 L diethyl ether (at room temperature, three days) -Evaporation of n-hexane under a vacuum using a rotary evaporator gave a diethyl ether extract of flowers. -The same protocol was used on 400 g of seeds air-dried for two weeks -Ground to a fine powder. -The third maceration with ethyl acetate three times (3 L, at room temperature for three days) gave ethyl acetate extract of seeds. (5–100 μg/mL)	in vitro	Genistin Taxifolin Quercetin Genistein 6-Methoxykaemferol Kaempferol Apigenin	Antioxidant activity (EAS IC_50_ = 15.13 μg/mL compared to BHT IC_50_ = 30.21 μg/mL) Enhance SIRT1 and SIRT3 genes expression in HaCaT cell anti-aging process in human keratinocytes	[3]
Flower Seeds Stems	-Obtain crude extracts of seeds, stems, flowers with methanol 70% -Fractionation by toluene, chloroform, ethyl acetate, butanol (7–10 mg/mL)	in vitro	Total phenolic Flavonoid compounds Condensed Tannins	Antioxidant activity Ethyl acetate extracts from seeds (IC_50_ = 0.15 ± 0.11 mg/mL)	[13]
	-The plant samples were air-dried for several weeks -Powdered seeds, stems and flowers -Maceration with methanol three times -Concentrated under reduced pressure -Fractionation with equal volumes of three organic solvents (hexane, dichloromethane, ethyl acetate) -Evaporated Fractions to dryness under vacuum and stored at +4°C -A concentration of 500 mg/mL was prepared by reconstituting the crude extracts in absolute methanol (500 mg/mL)	in vitro	Alkaloids Tannins Flavonoids Saponosides Terpenoids Coumarines	Antibacterial activity against: *Bacillus sp* *B. cereus* *Listeria ivanovii* *Staphylococcus aureus* *C. freundii* *E. coli* *Salmonella sp*	[14]
Leaves	-Extracted successively using a Soxhlet apparatus with n-hexane and methanol -Obtain hexane and methanolic extract -Evaporated by a Rotavapor to give dried extracts -Extracted with dichloromethane and ethyl acetate to obtain dichloromethane and ethyl acetate fractions (5–80 μg/mL)	in vitro	α-Pinene 1,8-Cineole 9H-pyrrolo [3′,4′:3,4]pyrrolo [2,1-a]phthalazine-9, 11(10H)-dione,10-ethyl-8-phenyl Sparteine Hexadecanoic acid L methyl cytisine 17- oxosparteine 4-(N-(3-trifluoromethylphenyl)-amino)-5,6 dimethyl-7H-pyrro [2.3-d]pyrimidine Lupanine Anagyrine	Antiproliferative effects on human cervical cancer cells	[30]
	-Extracted by using a Soxhlet apparatus with n-Hexane (1.3 L) and methanol (1.3 L) to obtain hexane and methanolic extract -Evaporated by a Rotavapor to give dried extracts -Extracted with dichloromethane (1.3 L) and ethyl acetate (1.3 L) to obtain dichloromethane fraction and ethyl acetate fraction (1–50 μg/mL)	in vitro	Quinolizidine Alkaloids Sparteine L-methyl cytisine 17-Oxosparteine Lupanine Anagyrine	Antitumoral activity (Rm-DF) was the most active extract, with a significant cytotoxic activity on HeLa and SiHa cells in a dose-dependent manner	[32]
		in vitro	Palmitic Acid trimethyl silyl ester Aphylline Phytol Linoleic Acid trimethyl silyl α Linoleic Acid trimethyl silyl ester Octadecanoic Acid, trimethyl silyl ester oleamide/SLEEPAMIDE Eicosanoic Acid, trimethylsilylester Monolupine/anagyrine Hexadecanoic Acid 2,3-bis[(trimethylsilyl)oxy]propyl ester dodecanoic Acid trimethylsilyl ester 1-Tetracosanol Stearic Acid 2,3-bis(trimethylsilyloxy)propyl ester Hexacosanoic Acid Stigmasterol trimethylsilylether β-Sitosteryltrimethylsilylether Campesterol	Anti-leukemic activity Cell cycle arrest and cell death occur through extrinsic apoptosis	[4,32]
Aerial parts	-Dried aerial parts at room temperature. -Powdered 200 g of plant material -Extracted with water (400 mL) at 70 °C, under agitation using a magnetic stirrer, during 1 h -The water extract was lyophilised to provide a crude water extract with 14 % yield (28.7 g). (18 mg/kg) (9 mg/kg)	in vivo Crohn’s disease model: -Aqueous extract of *R.* *monosperma* (9–18 mg/kg p.o.) was suspended in 0.9 % saline solution -Administered by gavage 48, 24 and 1 h prior to the induction of colitis and 24 h later. -Control group received a vehicle in a comparable volume (10 mL/kg animal). -The rats were checked daily for behavior, body weight and stool consistency. -Finally, animals were killed, using an overdose of chloral hydrate 48 h after induction of colitis	Daidzin Rutin Genistin Daidzein Luteolin Apigenin Genistein	Anti-inflammatory effects reduced the inflammation and damage in (TNBS)-induced colonic mucosa	[16]
Stems Leaves Flowers Seeds	-Extracted with absolute methanol three times at room temperature -Filtration -Combined methanolic extracts and concentrated to dryness under reduced pressure (31.25–500 µg extract/mL DMSO)	in vitro	Sparteine Dehydrosparteine β-Isosparteine Ammodendrine N-Methylcytisine Dehydrocytisine Cytisine 17-Oxosparteine Isolupanine 5.6-Dehydrolupanine 11.12-Dehydrolupanine Anagyrine Thermopsine	Antifungal activity against: *C. albicans* *C. tropicalis* *A. niger*	[17]

## Data Availability

No new data were created or analyzed in this study. Data sharing is not applicable to this article.
